# Catastrophic care-seeking costs as an indicator for lung health

**DOI:** 10.1186/1753-6561-9-S10-S4

**Published:** 2015-12-18

**Authors:** SB Squire, Rachael Thomson, Ireen Namakhoma, Asma El Sony, Afranio Kritski, Jason Madan

**Affiliations:** 1 of Clinical Sciences, Liverpool School of Tropical Medicine, Pembroke Place, UK; 2 REACH Trust, Lilongwe, Malawi; 3 EpiLab, Khartoum, Sudan; 4 Federal University of Rio de Janeiro, Brazil; 5 Division of Health Sciences, Warwick Medical School, University of Warwick, UK

## Abstract

Costs incurred during care-seeking for chronic respiratory disease are a major problem with severe consequences for socio-economic status and health outcomes. Most of the published evidence to date relates to tuberculosis (TB) and there is a lack of information for the major non-communicable chronic respiratory diseases: asthma and chronic obstructive pulmonary disease (COPD). International policy is recognising the need to address this problem and measure progress towards eliminating catastrophic care-seeking costs (see the post-2015 TB strategy). Current tools for measuring, defining, and understanding the full consequences of catastrophic care-seeking costs are inadequate. We propose two areas of work which are urgently needed to prepare health systems and countries for the burden of chronic lung disease that will fall on poor populations in the coming 10-20 years:

a) Rapid scale up of the number and scope of studies of patient costs associated with chronic non-communicable respiratory disease.

b) Work towards deeper understanding and effective measurement of catastrophic care-seeking costs.

This will produce a range of indicators, such as dissaving, which can more effectively inform health policy decision-making for lung health. These will also be useful for other health problems. We argue that reduction in care-seeking costs will be a key monitoring indicator for improvements in lung health in particular, and health in general, in the coming 10 to 20 years.

## Introduction

The term ‘catastrophic costs’ is commonly used to refer to healthcare costs that place excessive burdens on patient households. Health expenditure is said to be catastrophic if it ‘threatens a household's ability to meet its subsistence needs[1]. This is a useful concept in helping to set a benchmark within the overall health systems strengthening goal of social and financial risk protection. Health systems should function in such a way as to minimise the extent to which patients and their households or families have to use their own private resources (out-of-pocket expenditure) to finance health care seeking activities. This is not only a matter of providing Universal Health Coverage to reduce direct medical costs (such as consultation fees, costs of laboratory tests and imaging, and drugs), but also a matter of paying attention to the other building blocks of health systems so that health services are provided in ways that enable patients to follow simplified care-seeking pathways. Simplified care-seeking pathways require fewer health facility visits and their attendant direct non-medical costs (such as food and transport) and indirect costs (lost income).

While useful as a concept, catastrophic costs are problematic in terms of precise definition, understanding and accurate measurement. Minimisation of catastrophic costs would be an attractive indicator of effective health system function, but patient costs are not measured systematically on a wide scale in routine health service delivery, and are difficult to bench mark against either individual or household income. This is an issue for all health service delivery, and particularly for chronic disease management which requires long term and repeat engagement with health services. Chronic diseases are predicted to be an increasing problem over the coming 10-20 years in developing countries. Within this large category of disease, chronic respiratory disease will be a particular problem amongst poor populations (see CAHRD Papers LH Biomass, Cough). In this paper, therefore, we assess the current state of knowledge about patient costs and catastrophic costs in chronic respiratory disease. We present ideas on how this knowledge informs plans for the essential future work that is required to develop approaches to the measurement, understanding, and use of catastrophic costs in evaluating effectiveness of lung health interventions in particular and health systems in general.

## Evidence for the magnitude of the problem of care-seeking costs in chronic lung disease

Over the past 20 years there have been a number of studies from across the globe highlighting that TB patients incur substantial costs during care seeking in low and middle income countries. These studies have recently been brought together into a single systematic review(1). There have also been two previous systematic reviews of relevant studies from Africa[3,4]. In the most recent review(1)], 49 studies met the inclusion criteria and costs were stratified into direct medical costs (consultations, tests, medicine, hospitalisation, etc) direct non-medical costs (transport & food during health care visits etc) and indirect costs (lost income). Costs were also reported as a percentage of annual income expressed in a number of different ways. Key results are summarised in Table 1.

**Table 1:** Patient costs as a percentage of annual income (average of mean) from[2]

	Studies n	Direct costs %	Lost income %	Total cost %
**Individual**				

Reported income	22	21	37	58

Annual Wage^2^	35	9	21	30

Wage of lowest 20%^3^	34	25	64	89

**Reported household income**	7	16	22	39

^2^ Computed from “gross average nominal monthly wage in the International Labour Organisation's global wage database http://www.ilo.org/travil/info/db/lang--en/index.htm

^3^ World Bank. Income Share Held by Lowest 20% in the World Bank Database http://data.workdbank.org/indicator/SI.DST.FRST.20/countries

Depending on the type of costs reported, costs varied from a small fraction of mean monthly income for average annual income earners to over 10 times the annual income that the average person in the income-poorest 20% of the population earns. It is striking that these impoverishing expenditures have been recorded even in health systems which provide services free at the point of delivery (ie no user-fees)[5]. This indicates that even when a health system provides universal health coverage, care-seeking costs can be catastrophic.

**Figure 1:**
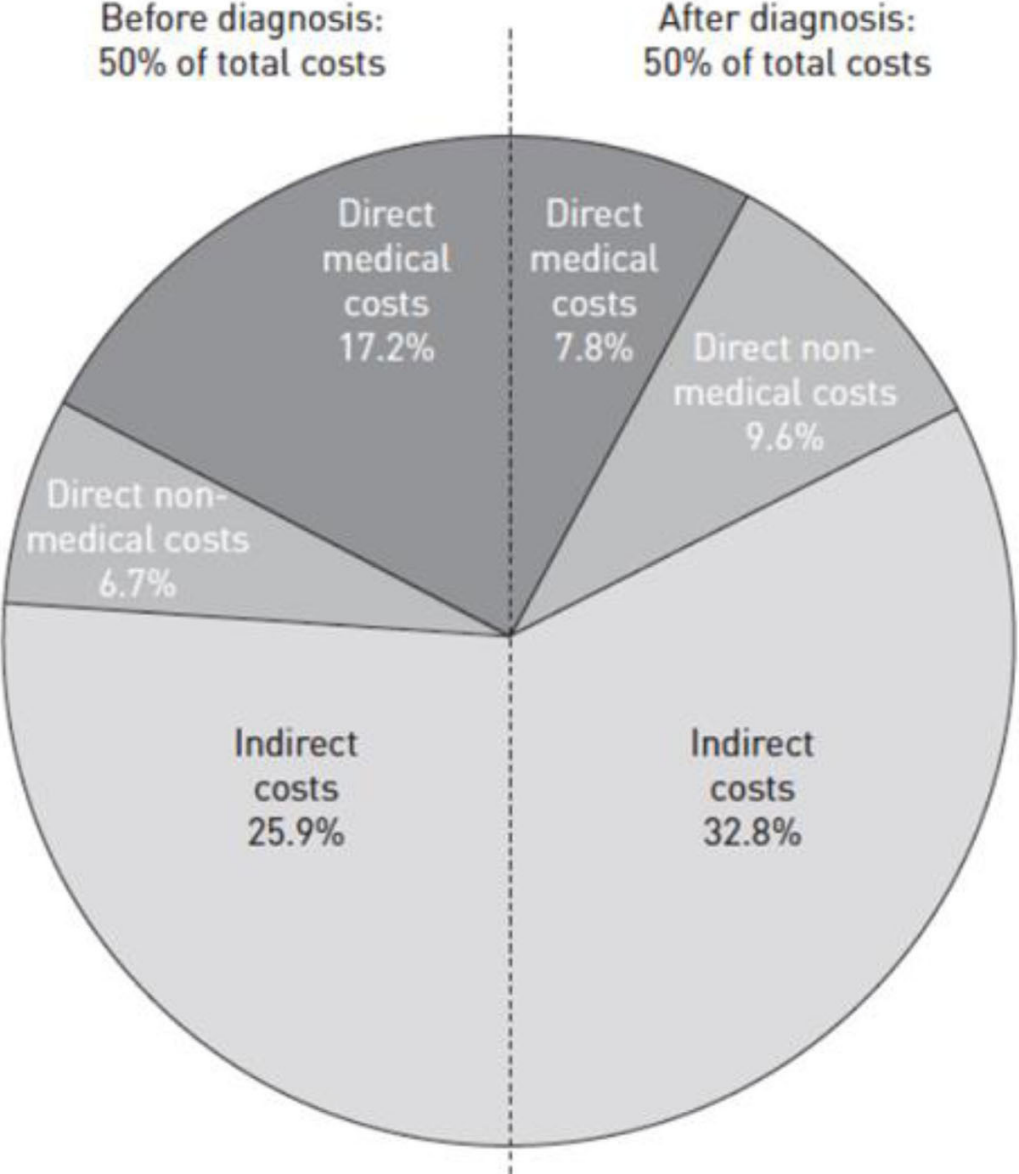
Breakdown of direct and indirect costs before and during treatment (8 studies) (1)

In the systematic review the cost components were extracted separately for pre- and post-TB treatment periods and, in broad terms, 50% of total costs were incurred during care-seeking before diagnosis as illustrated in Figure 1.

By contrast with this wealth of knowledge for TB, there is a marked lack of published studies from low and middle income countries on the patient costs associated with the major non-communicable lung diseases – asthma and chronic obstructive airways disease (COPD). Only one study has documented the cost of inhaled corticosteroids for asthma(2)]. It surveyed the prices and policies for components of asthma treatment in 1998 in Algeria, Burkina Faso, Ivory Coast, Guinea, Mali, Syria, Turkey and Vietnam and documented that in all but two countries, the cost of one year of drugs for treatment of a moderate, persistent asthma case exceeded the monthly salary of a nurse in that country. We were unable to find published information on costs incurred in care-seeking by asthma patients. One study from China on COPD patients includes some data on patient costs and documents that the mean yearly direct economic burden of one COPD patient accounted for an average of one third of family income(3)]. One modeling study of the cost-effectiveness of asthma and COPD interventions in Sub-Saharan Africa and South East Asia ranks low dose inhaled corticosteroids for mild persistent asthma as the most cost-effective of the interventions studied[8]. However the source for the patient costs used in the model is not clear.

## Evidence of the impact of catastrophic care-seeking costs on socio-economic status and health outcomes

In the TB literature to date, various possible definitions of “catastrophic” costs have been proposed: “>10% of monthly household income”(4)], “>10% of annual household income(5)] and[11], >40% of non-subsistence household income[12] and[13]. WHO suggests that “catastrophic health expenditure” should be defined as “direct healthcare expenditures corresponding to >40% of annual discretionary income (income after basic needs, such as food and housing[14]. Whichever definition is adopted, catastrophic health expenditures lead to reduced food consumption, taking children out of school, taking out loans, and selling assets. Each of these coping mechanisms leads to further impoverishment of individuals and households and the establishment of a vicious and progressive negative interaction between TB and poverty.

Pre-diagnostic costs, which are usually the costs incurred in care-seeking for patients who have chronic cough, (not just TB) are also obstacles which are difficult for poorer patients to overcome. In this way, these costs also contribute to the problem of drop-out between the start of care-seeking, the initiation of TB treatment and successful cure. While factors, including patient costs, which affect drop-out after the start of TB treatment have received much attention(6), the problem of drop-out between the start of care-seeking and the start of TB treatment has been less studied. This has partly been rectified by a recent systematic review which has quantified the drop-out between diagnosis and the start of treatment(7) to be around 15%. The problem of drop-out between the start of care-seeking and diagnosis is more difficult to study because

a) there is considerable difficulty in defining and capturing the start date of care seeking or onset of symptoms, and

b) care seeking costs at this stage are incurred for all chronic cough patients even though fewer than 20% will end up with a diagnosis of TB.

It is likely that drop out for this stage is at least as large as drop-out for the stage between diagnosis and the start of treatment. For both stages, drop-out is likely to be exacerbated when patients face substantial care-seeking costs and will lead to poor health outcomes for all patients with chronic respiratory disease.

There are also wider consequences than the direct effects on patients themselves. For TB patients there is the added public health consequence of leaving patients untreated and acting as sources of on-going transmission of infection. For asthma patients and their families there are the costs of emergency room treatment for acute exacerbations that could have been averted by coherent diagnostic and treatment services (see CAHRD Paper LH Cough).

## Recognition of the importance of catastrophic costs in international policy: the post 2015 STOP-TB Strategy

Prior to 2015, none of the strategic TB control frameworks (DOTS, the Stop TB Strategy and the Global Plan to Stop TB 2006–2015) included a specific target on poverty. This has now changed. On 19th May 2014, the World Health Assembly endorsed a strategy which includes a target that “*no families will face catastrophic costs due to TB by 2020”*. Achievement of this target is to be maintained through to 2035 (see Table 2) &http://www.who.int/tb/post2015_TBstrategy.pdf?ua=1

**Table 2:** Key global indicators, milestones and targets for the post-2015 tuberculosis strategy

Indicators with baseline values for 2015	Milestones			Targets
	
	2020	2025	2030	2035
*Percentage reduction in deaths due to tuberculosis* *(projected 2015 baseline: 1.3 million deaths)*	35%	75%	90%	95%

*Percentage and absolute reduction in tuberculosis incidence rate* *(projected 2015 baseline 110/100 000)*	20% (<85/100 000)	50% (<55/100 000)	80% (<20/100 000)	90% (<10/100 000)

*Percentage of affected families facing catastrophic costs due to tuberculosis^1^* *(projected 2015 baseline: not yet available)*	Zero	Zero	Zero	Zero

^1^The fact that there is no baseline from which to work is indicative of the problems faced in measurement of this target.

The post-2015 TB strategy contains a strong focus on measures to promote the attainment of this target within the TB community (the development of patient-centred care), within the health sector (progress towards universal health coverage) and beyond the health sector (social protection).

For TB at least, meaningful, evidence-based indicators of catastrophic costs which can be used at scale will be needed. Given predictions about the rise in non-communicable respiratory disease, such indicators are likely to become increasingly important for all patients with chronic respiratory disease. Although patient questionnaires (see below) to measure these indicators have been developed and used in TB, there is little experience of their use for other chronic respiratory disease and they are far from being fit for purpose.

## Problems with defining catastrophic costs and developing practical indicators

In practice, catastrophic costs are commonly defined as a threshold percentage of income or usual expenditure. There is, however, considerable debate as to the appropriate threshold to use to define costs as catastrophic (see above). There are problems defining and measuring *discretional* expenditure and the threshold for catastrophic *total* cost (including indirect costs) has not yet been established.

Calculating patient costs involves completing lengthy and complex patient cost questionnaires. Early iterations of such questionnaires[8,9] were further developed by the TB & Poverty Subgroup of The STOP-TB Partnership into a patient costing tool http://www.stoptb.org/wg/dots_expansion/tbandpoverty/spotlight.asp). Even though considerable efforts were made to make this tool as user-friendly as possible, experience of its use in a number of settings[10] and[11] indicates that it is too cumbersome for routine use in monitoring and evaluation. In addition it is subject to recall bias.

There are well known methodological challenges in the identification and measurement of indirect costs. Similarly, accurately quantifying income in the most disadvantaged patient groups is challenging. Such patients will often not receive a regular cash income, but rely on a range of activities generating cash and in-kind income that is irregular and subject to seasonal fluctuations. For this reason, academic research often relies on indirect measurement of income via socioeconomic indicators. This also involves complex questionnaire-based analyses of, for example, asset ownership. Estimation of catastrophic costs in terms of cost/income ratios is therefore a significant difficulty in research projects, and may be unfeasible in routine monitoring of policy and practice.

## Implications of work on care-seeking costs in TB for chronic respiratory disease

To date the literature on care-seeking costs has been based on research amongst patients who have achieved a diagnosis of TB and has involved asking them to recall their experiences leading up to diagnosis and through treatment. There are several problems with this situation. First there is the problem of recall bias (see above). Second it means that there is a dearth of information about care-seeking costs amongst TB patients who never achieve a diagnosis. For most TB patients, care seeking starts with the development of a persistent, chronic cough. This is a common symptom affecting 5-10% of the adult population in developing countries[12]. Although primary health care services are alerted to the importance of testing for TB in patients with chronic cough, we know that even in high burden TB countries fewer than 20% of such patients will have TB[19]. Information on the nature of disease amongst the remaining 80% of patients is scarce. There is, however, increasing information suggesting that not all of this disease burden is due to infectious agents; non-communicable airways disease (asthma, bronchiectasis and chronic obstructive pulmonary disease) is of increasing importance. There are indications that patients with chronic cough who are not diagnosed with TB have poor outcomes[13] suggesting that care provision for them is inadequate. In many settings, HIV co-infection will also influence care-seeking behaviour and costs both for those with TB and those without. It is clear that the burden of non-communicable respiratory disease will rise inexorably in developing countries over the next 10-20 years and risk factors for these, such as exposure to indoor air pollution (see CAHRD Paper LH Biomass) and tobacco smoke are also risk factors for TB. Finally, the chronicity and disability that are associated with COPD and asthma are additional factors which are likely to escalate the burden of patient costs.

Overall, therefore, there is a clear need to broaden investigation of care-seeking costs from patients with TB to include patients with undifferentiated chronic cough and to work from there towards understanding and reducing these costs for non-TB causes of chronic cough.

## A way forward

We propose two initial areas of work which are urgently needed to prepare health systems and countries for the burden of chronic lung disease that will fall on poor populations in the coming 10-20 years:

*A. Rapid scale up of the number and scale of studies of patient costs associated with chronic non-communicable respiratory disease*.

The first step should be the conduct of a systematic review of the existing literature in this field. This would serve to better understand the gaps in the knowledge about these costs and the tools that have been used to measure them. Following the systematic review, there should be a series of studies which could use modifications of the data collection tools that have been developed for TB, where the modifications are informed by the findings of the systematic review.

*B. Work towards deeper understanding and effective measurement of catastrophic care-seeking costs*.

**Figure 2:**
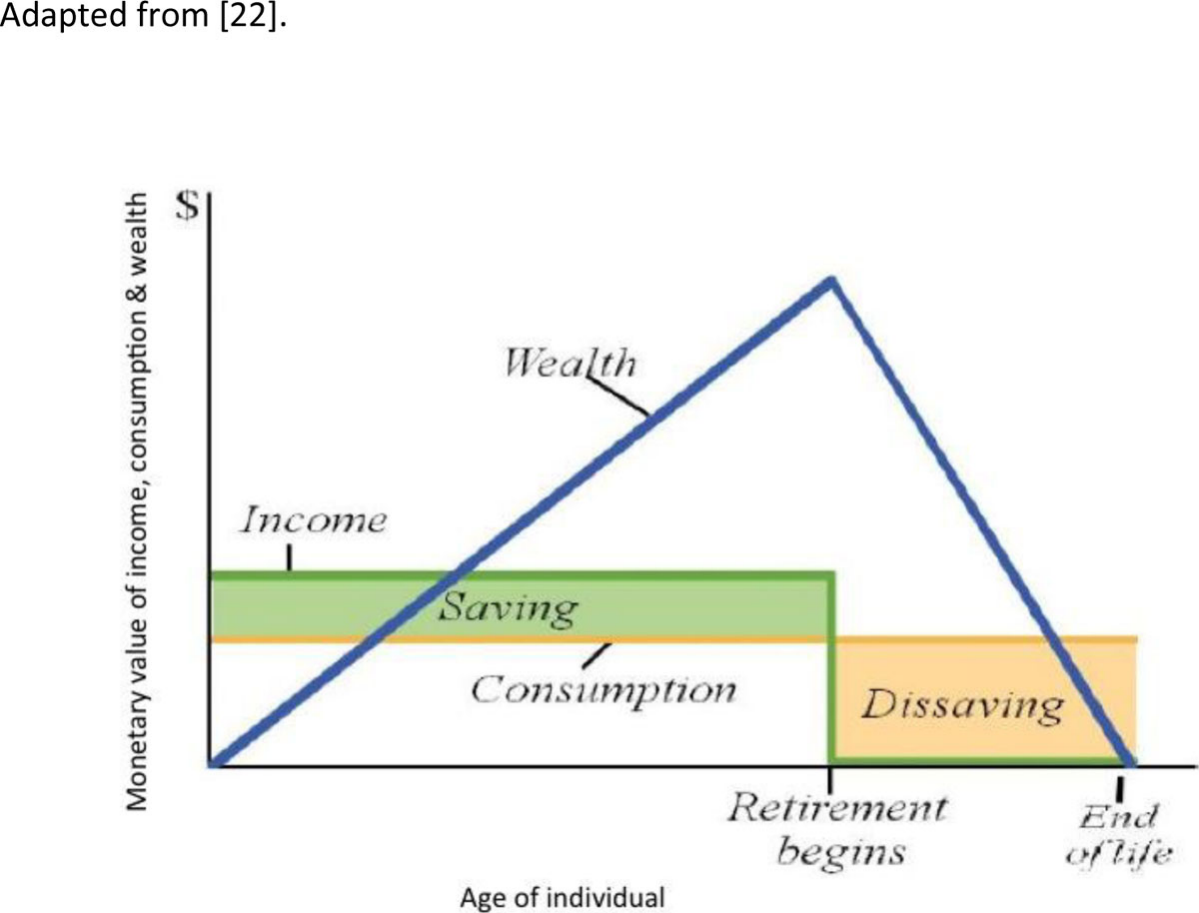
Dissaving as an economic concept Adapted from [22].

We suggest an alternative approach to the questionnaire-based data collection and analysis approaches which have been used to date and described above. This new approach focuses on the sacrifices households make to finance healthcare expenditure. These may include reduction in consumption, withdrawing children from school, and so on. A common coping strategy is to draw on the financial resources available to the household for consumption smoothing, either by disposing of assets or taking out loans. We refer to this strategy as ‘*dissaving*’, as it involves reducing the financial resources available to protect the household from further shocks. It is effectively the opposite of ‘*saving*’, which strengths the financial resources of the household (see Figure 2). Dissaving can easily and quickly be measured by surveys, and is likely to be correlated with excessive costs. A survey of households in 40 developing countries found that 1 in 4 borrowed money or sold assets to fund healthcare costs[21], and that the poorest households were most likely to dissave. However, there is limited research on the exact relationship between dissaving and catastrophic healthcare expenditure, the importance of the type of asset being sold or the source of loan finance, and the impact of dissaving on household wellbeing.

A theoretical model of household decision-making when seeking health care under financial pressure would be a useful guide for the design, evaluation and selection of both social protection and health interventions for patients with chronic respiratory disease. A number of decision-making models have been developed within the economics literature, based on Grossman's work on health capital production [24] Models have been developed that treat households as single entities, as multiple individuals making cooperative decision to jointly produce household health and as multiple decision-makers with independent (and possibly conflicting) goals[14,15]

While such models have been widely used in high-income country settings to model choices such as smoking, vaccination take-up, and childhood obesity, research on their use and effectiveness in low and middle income settings is limited. The assumption of rational decision-making which underpins these models may turn out to be unrealistic and over-simplistic. Theoretical decision models drawing on other social sciences, that incorporate concepts such as behavioural norms, gender analysis and cultural influence, may provide an approach more consistent with observed behaviour.

We propose a programme of empirical research to collect evidence on the impact on households of costs associated with seeking health care for chronic respiratory disease, the actions taken by household members in response to this financial stress, the consequences of those actions for household health and well-being, and the decision-making process behind household actions. The aims of this research are two-fold. Firstly, we aim to identify and validate indicators (such as dissaving) for catastrophic health care expenditure in its underlying sense – that of resulting in long-term significant impoverishment of those affected. These indicators should be simple enough for use in routine monitoring and evaluation, and validated by our research as indicative of severe and lasting financial distress. Secondly, we aim to evaluate and refine alternative theories of household decision-making to identify models consist with actual behaviour in low-income settings. We anticipate that validated theoretical models will be extremely useful for policy-makers in the selection and design of interventions around social protection, poverty reduction, and universal health care implementation.

The proposed programme of research will draw on a range of qualitative and quantitative methods to provide evidence that is robust and captures the complex decision-making context faced by poor households under health-related financial pressures. It will be carried out in low and middle income countries and integrated with intervention studies aimed at reducing catastrophic costs and improving access to health services. It will draw on relevant applied and methodological expertise across LSTM and the University of Warwick, as well as the network of in-country research organisations where links have already been established. In addition, capacity to undertake this work and use it to influence delivery and policy will need to be strengthened in existing and new in-country collaborating organisations.

## Conclusions

Costs incurred during care-seeking for chronic respiratory disease are a major problem with severe consequences for socio-economic status and health outcomes. Most of the published evidence to date relates to tuberculosis (TB) and there is a lack of information for the major non-communicable chronic respiratory diseases, asthma and chronic obstructive pulmonary disease (COPD). Current tools for measuring, defining, and understanding the full consequences of catastrophic care-seeking costs are inadequate. Two areas of work are urgently needed to prepare health systems and countries for the burden of chronic lung disease that will fall on poor populations in the coming 10-20 years:

*a) Rapid scale up of the number and scope of studies of patient costs associated with chronic non-communicable respiratory disease*.

*b) Work towards deeper understanding and effective measurement of catastrophic care-seeking costs*.

This will produce a range of indicators, such as *dissaving*, which can more effectively inform health policy decision-making for lung health. They may also be useful for other health problems. Reduction in care-seeking costs will be a key monitoring indicator for improvements in lung health in particular, and health in general, in the coming 10 to 20 years.

## Competing interests

The authors declare they have no competing interests.

## Authors' contributions

All authors contributed to the development of the final manuscript.

## Declarations

This article has been published as part of *BMC Proceedings* Volume 9 Supplement 10, 2015: The 2014 CAHRD (Collaboration for Applied Health Research & Delivery) Consultation. The full contents of the supplement are available online at http://www.biomedcentral.com/bmcproc/supplements/9/S10.
